# Outcome of conservative treatment for triangular fibrocartilage complex lesions with stable distal radioulnar joint

**DOI:** 10.1007/s00068-020-01315-2

**Published:** 2020-02-08

**Authors:** Anna Lena Sander, Katharina Sommer, Antonia Katharina Kaiser, Ingo Marzi, Johannes Frank

**Affiliations:** Department of Trauma, Hand and Reconstructive Surgery, University Hospital Frankfurt, Theodor-Stern-Kai 7, 60590 Frankfurt am Main, Germany

**Keywords:** TFCC lesion, Conservative treatment, Arthroscopic debridement, Stable DRUJ

## Abstract

**Purpose:**

Triangular fibrocartilage complex (TFCC) lesions have high clinical relevance. Although multiple studies have been done in the past, there is a lack of data after conservative treatment and controversy remains regarding management. The purpose of this study was to compare the outcome of symptomatic TFCC lesions after conservative treatment and arthroscopic debridement.

**Methods:**

Between 2012 and 2017, 33 patients were included. 16 patients were treated conservatively and 17 patients with arthroscopic debridement. The average age was 41 years (range 18–63). The mean follow-up was 22.2 months (range 6–74). Evaluation included pain, range of motion (ROM), grip strength, Disabilities of the Arm, Shoulder, and Hand (DASH) score, Modified Mayo Wrist Score (MMWS), and Purdue Pegboard test.

**Results:**

Pain averaged 0.1 (range 0–1) in the conservative group compared to 1.3 (range 0–6) in the arthroscopic group. The mean ROM was 99% for wrist extension, and 100% for flexion and pro-/supination in the conservative group, and 96% for extension and flexion, and 100% for pro-/supination in the arthoscopic group compared to the contralateral side. Grip strength was 88% (range 63–100) in the conservative group versus 89% (range 33–100) in the arthroscopic group. The conservative group reached a DASH score of 16.8 and MMWS of 94.3 compared to 22.1 and 87.2 in the arthroscopic group. The differences were not statistically significant.

**Conclusions:**

Our study demonstrated similar results of conservative compared to arthroscopic treatment. Because conservative treatment was a sufficient and reliable option, we propose it as first-line treatment for TFCC lesions with stable distal radioulnar joint.

## Introduction

Injuries and degenerative changes of the triangular fibrocartilage complex (TFCC) are a common cause of ulnar-sided wrist pain and disabilities [1–3]. Despite improvement in understanding and classification of these lesions, controversy remains regarding management and evidence-based recommendations for the treatment of TFCC lesions could not be concluded from literature [4]. In this context, it is also evident that there is a lack of data after conservative treatment. While this can be caused by one-sided publication strategies, it reflects operative treatment as current standard procedure [4]. The purpose of this study was to determine subjective and objective outcome of conservative treatment and arthroscopic debridement of patients with symptomatic TFCC lesions and stable distal radioulnar joint (DRUJ) hypothesising comparable satisfactory results.

## Patients and methods

### Patients

Approval from the institutional review board of the medical faculty (GN481/15) was obtained prior to performing this study. A retrospective analysis was performed on a cohort of patients aged 18–65 years with TFCC lesions between February 2012 and March 2017.

The inclusion criteria in this study were (1) TFCC lesion, (2) positive results to provocative tests such as the ulnocarpal stress test and ulnar fovea sign test, (3) stable DRUJ, and (4) conservative treatment or arthroscopic debridement as chosen treatment [5]. The exclusion criteria were (1) arthroscopic suture of the TFCC, (2) instability of the DRUJ, (3) ulnocarpal impaction syndrome, (4) scapholunate or lunotriquetral ligament injury, and (5) advanced osteoarthritis of the radiocarpal joint. The ulnocarpal impaction syndrome can also develop in wrists with neutral or negative ulnar variance [6]. We therefore excluded patients with ulnocarpal impaction syndrome regardless of ulnar variance.

133 patients with TFCC lesions were identified. 20 patients were excluded due to the exclusion criteria. 80 patients were lost to follow-up due to the following reasons: (1) the patient did not want to participate in the study, (2) the contact information was no longer correct, (3) the patient did not come to the follow-up appointment, and (4) the patient was not able to participate in the study due to long journey.

A total of 33 patients could be examined for follow-up. 16 patients were treated conservatively and 17 patients with arthroscopic debridement. The ratio between men and women was 18:15. In the conservative group, the ratio was 11:5 compared to 7:10 in the arthroscopic group. The average age was 41 years (range 18–63). In the conservative group, the average age was 45.4 years (range 23–61) versus 36.8 years (range 18–63) in the arthroscopic group. In the conservative group, the dominant hand was affected in 50% compared to 71% in the arthoscopic group.

Results of magnetic resonance imaging (MRI) of the wrist were available in all cases. The TFCC lesions were diagnosed by T1- and T2-weighted MRI. In the arthroscopic group, the TFCC lesions were classified according to Palmer revealing 6 type 1A, 5 type 1B, 3 type 1D, 2 type 2A, and 1 type 2B [7]. The mean period of follow-up was 22.2 months (range 6–74) (Table 1).

**Table 1:** Patients

	Conservative group	Arthroscopic group
Number of patients	16	17
Sex (male/female)	11/5	7/10
Age (years)	45.4 (23–61)	36.8 (18–63)
Dominant hand (%)	50	71
Classification		
1A		6
1B		5
1D		3
2A		2
2B		1
Follow-up (months)	22.2 (6–74)	

### Clinical assessment

DRUJ instability was evaluated through passive anteroposterior translation of the ulna on the radius, while the forearm was positioned in neutral, full pronation, and full supination. The test was positive if the ulna displaced 1 cm or more relative to the contralateral side without clear endpoint resistance [5, 6, 8, 9].

Wrist pain was assessed using a visual analogue scale (VAS 1–10). Pain experienced during manual use was defined as exertional.

Range of motion (ROM) was measured with a standard goniometer. The ROM included extension/flexion of the wrist, and pro-/supination of the forearm. The results were reported as percentages of the contralateral wrist.

Grip strength was determined by a BASELINE^®^ Hydraulic Hand Dynamometer (Fabrication Enterprises, Inc., White Plains, New York, USA) at level 2. Each hand was measured three times and the mean values were calculated. Grip strength data were reported as percentages of the contralateral side.

The Disabilities of the Arm, Shoulder, and Hand (DASH) score (0: no limitation, 100: maximum limitation) and the Modified Mayo Wrist Score (MMWS; 91–100: excellent, 80–90: good, 65–79: fair, less than 65: poor) were used to evaluate functional outcome [10–12].

Manual finger and hand dexterity were assessed by the Purdue Pegboard test with three test trials per subtest [13].

### Conservative treatment

A forearm orthosis was applied for 2 weeks maintaining the wrist in slight extension. Afterwards, the orthosis was recommended at night-time and if required. Full weight-bearing was allowed after a total period of 6 weeks. Additionally, conservative treatment included nonsteroidal anti-inflammatory medication and physical therapy. 3 patients received a corticosteroid and local anaesthetic injection (triamcinolone and ropivacaine, 2 ml).

### Arthroscopic debridement

**Fig. 1:**
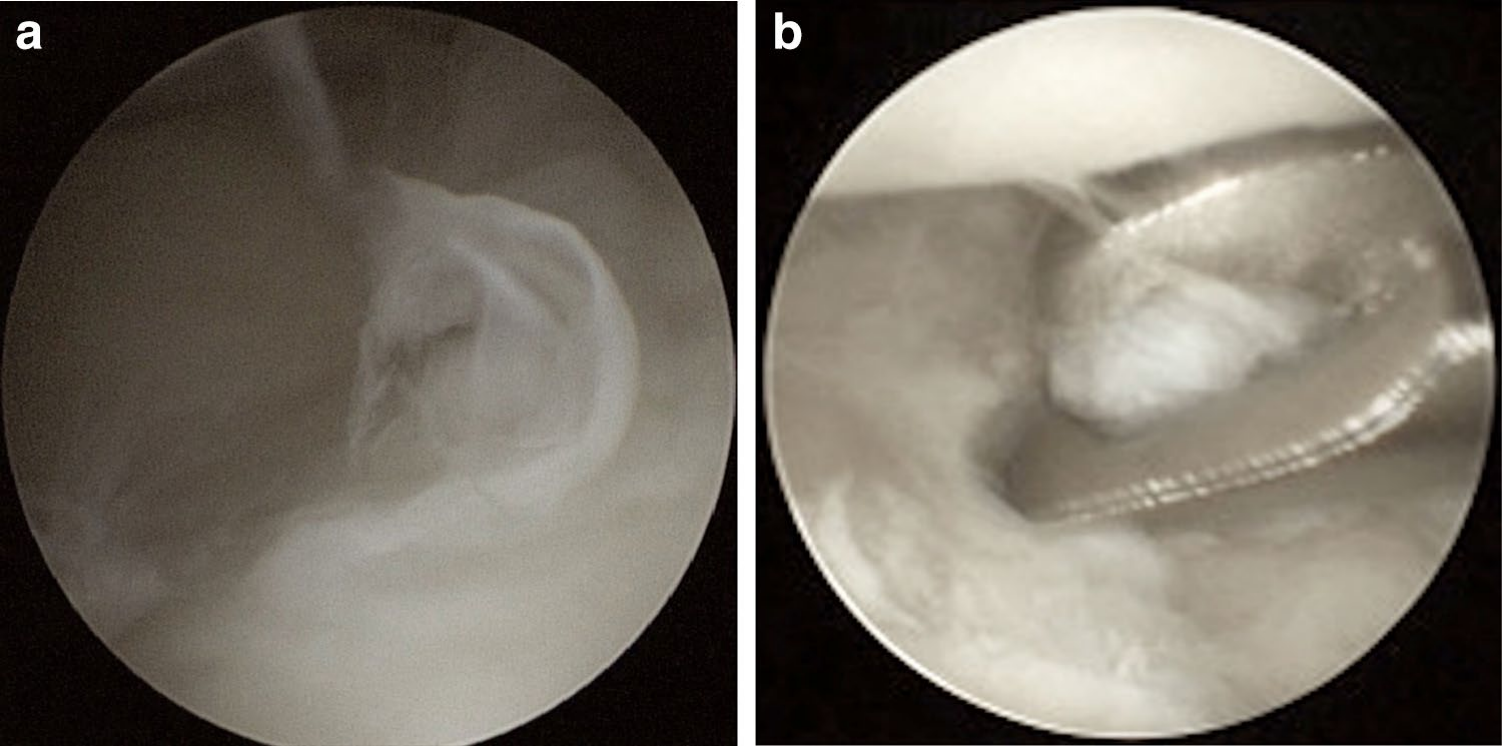
Arthroscopic view of the TFCC. **a** TFCC Palmer type 1A lesion. **b** Arthroscopic debridement

The operation was performed under regional or general anaesthesia. A pneumatic tourniquet was routinely used with a pressure of 280 mmHg. Wrist distraction (4 kg) was achieved by means of a wrist traction tower. A 2.7-mm arthroscope was introduced in accordance with the standard technique through the 3/4 and 6R portals. The integrity and stability of the TFCC and the intercarpal ligaments were assessed with the probe, and standardised tests (hook test, trampoline test) were performed [14, 15]. Any TFCC tears present were then classified according to Palmer [7]. The debridement of the TFCC lesions was performed using an arthroscopic punch, grasping forceps and shaver without violating the palmar and dorsal radioulnar ligaments (Fig. 1). All patients received a corticosteroid and local anaesthetic injection (triamcinolone and ropivacaine, 2 ml). A forearm cast was applied postoperatively for 2 weeks maintaining the wrist in slight extension. Afterwards, a forearm orthosis was recommended at night-time and if required. Full weight-bearing was allowed after a total period of 6 weeks.

### Statistical analysis

Statistical evaluation was performed using Mann–Whitney test and Chi-square test. Values of *P* < 0.05 were considered statistically significant.

## Results

In reponse to the DRUJ instability test, all patients in both groups demonstrated a stable DRUJ during the clinical examination before and after treatment.

For the conservative group, postoperative pain symptoms averaged 0.1 (range 0–1) at rest and 1.8 (range 0–4) on exertion. For the arthroscopic group, pain was evaluated with 1.3 (range 0–6) at rest and 3.2 (range 0–8) on exertion. No significant differences were found between the conservative and operated wrists.

The average ROM of the conservative group was 99% for wrist extension, and 100% for wrist flexion and forearm pro-/supination when compared with the contralateral side. In the arthroscopic group, wrist extension and flexion averaged 96%, and 100% for forearm pro-/supination. The differences were not statistically significant.

Grip strength was 88% (range 63–100) in the conservative group versus 89% (range 33–100) in the arthroscopic group compared to the contralateral side. The conservative and arthroscopic group did not differ significantly regarding grip strength.

At the final examination, the mean DASH score averaged 16.8 in the conservative group compared to 22.1 in the arthroscopic group. The functional outcome evaluated by the MMWS was 94.3 (53% excellent, 47% good) in the conservative group, and 87.2 (56% excellent, 31% good, 13% poor) in the arthroscopic group. No significant differences were found between both groups.

In the Purdue Pegboard test, no significant differences were assessed in the subtests between the conservative and arthroscopic group (Table 2).

**Table 2:** Results

	Conservative group	Arthroscopic group
Positive DRUJ instability test (%)	0	0
Pain		
At rest	0.1 (0–1)	1.3 (0–6)
On exertion	1.8 (0–4)	3.2 (0–8)
ROM (% contralateral)		
Extension	99	96
Flexion	100	96
Pronation	100	100
Supination	100	100
Grip strength (% contralateral)	88 (63–100)	89 (33–100)
DASH score	16.8	22.1
MMWS	94.3	87.2
Excellent (%)	53	56
Good (%)	47	31
Poor (%)	–	13
Purdue Pegboard test		
Injured hand	13.1 (7.3–17)	12.7 (5.7–16.3)
Contralateral hand	12.4 (7.2–15.3)	13.1 (7.3–16)
Both hands	10.2 (6–12.7)	10.3 (5–13)
Assembly	24.8 (13.3–41.3)	24.9 (12–37.3)

## Discussion

Ulnar-sided wrist pain is one of the main problems of patients with TFCC lesions [16]. Arsalan-Werner et al. evaluated the clinical outcome of 43 patients with symptomatic TFCC Palmer type 1A lesions following arthroscopic debridement with a mean follow-up of 42.5 months. Motion pain and pain at rest improved significantly from 8.4 ± 1.6 to 2.6 ± 2.1, and 7.2 ± 2 to 1.4 ± 1.6, respectively [17]. Cardenas-Montemayor et al. determined functional and subjective results of 31 patients who received arthroscopic debridement for TFCC Palmer type 1B lesions with a mean follow-up of 26.7 months. There was a significant reduction in pain from 7.6 ± 1.7 to 2.3 ± 1.8 [18]. Möldner et al. investigated functional and subjective outcome parameters after arthroscopic debridement of 50 patients with TFCC Palmer type 2C lesions with a mean follow-up of 38 months demonstrating significant pain reduction from 7.6 to 2 [3]. Husby and Haugstvedt treated 35 patients with TFCC lesions by arthroscopic debridement at a median follow-up of 39 months. 8 patients were free of pain, 14 much better, 8 somewhat better, 5 unchanged, and no patient had got worse [19]. Osterman evaluated 41 patients treated arthroscopically for TFCC tears with an average follow-up of 23 months. 73% of patients demonstrated a complete relief of pain, 12% improved but continued to have intermittent symptoms, 10% reported no change from preoperative symptoms, and 5% got worse [20]. In our arthroscopic series, the results on the VAS were similar. Postoperative pain symptoms were determined with 1.3 (range 0–6) at rest and 3.2 (range 0–8) on exertion. Although conservative treatment averaged better values with 0.1 (range 0–1) and 1.8 (range 0–4), the differences were not statistically significant.

The degree of pronation and supination is a relevant indicator for the TFCC constitution and DRUJ stability [18, 21]. Arsalan-Werner et al. evaluated pronation with 98.4%, and supination with 95.6% of the contralateral side [17]. In the study by Cardenas-Montemayor et al., the postoperative pro-/supination ROM averaged 99.4% when compared with the contralateral wrist [18]. Möldner et al. as well as Husby and Haugstvedt recorded the pro-/supination sector as a median of 100% of the contralateral side [3, 19]. In the present study, the results of the arthroscopic group were congruent with a pro-/supination ROM of 100% when compared with the contralateral side. Averaging 100% in the conservative group, no significant difference was found between the conservative and operative group.

TFCC lesions are often associated with decreased grip strength and its adequate restoration is a primary treatment goal [16]. Arsalan-Werner et al. demonstrated a significant reduction of grip strength after surgery with 79.6% of the contralateral wrist [17]. In the study of Cardenas-Montemayor et al. grip strength was 96.7% of the contralateral side [18]. Husby and Haugstvedt recorded grip strength as a median of 94% [19]. Our results lied in between with a grip strength of 89% (range 33–100) in the arthroscopic group versus 88% (range 63–100) in the conservative group. The conservative and arthroscopic group did not differ significantly regarding grip strength.

An outcome measure which reflects the impact on function is a key component in the assessment of treatment success. In the study of Arsalan-Werner et al. the DASH score improved significantly from 49.8 ± 19.3 preoperatively to 14.1 ± 17.9 postoperatively. The mean MMWS was 78.8 ± 12.3 at follow-up. 53.5% of patients were rated excellent, 34.9% good, 7% satisfactory, and 4.7% poor [17]. Cardenas-Montemayor et al. measured a DASH score of 17.02 ± 14.92 and MMWS of 90 ± 9.1. The MMWS was rated excellent in 48% of patients, good in 39%, and fair in 13% [18]. Möldner et al. evaluated a DASH score of 18 and MMWS of 89 [3]. Husby and Haugstvedt assessed 13 patients as excellent, 14 good, 4 fair, and 1 poor [19]. Our results of the arthroscopic group were comparable with a DASH score of 22.1 and mean MMWS of 87.2. 56% of patients were excellent, 31% good, and 13% poor. In comparison, the DASH score was 16.8 and the average MMWS 94.3 in the conservative group. 53% of the patients were evaluated as excellent and 47% good. No significant differences were found between the conservative and operated wrists.

Some limitations must be considered for the present study. First, the study design was retrospective. Second, follow-up was only available for a relatively small number of patients (33/113), which, however, is consistent with current literature. Third, pain perception, DASH score, and MMWS data before treatment were lacking. We believe that a randomized blinded study that compares results of conservative treatment with arthroscopic debridement should be performed.

## Conclusion

Conservative treatment and arthroscopic debridement of TFCC lesions with stable DRUJ yielded comparable satisfactory to excellent results. Therefore, we believe that conservative treatment with immobilisation may be the first-line treatment for TFCC lesions with stable DRUJ, and arthroscopic debridement a therapeutic procedure after an intervall for TFCC lesions with persistent ulnar-sided wrist pain.

## References

[1] Atzei A, Luchetti R, Braidotti F (2015). Arthroscopic foveal repair of the triangular fibrocartilage complex. J Wrist Surg.

[2] Kirchberger MC, Unglaub F, Mühldorfer-Fodor M, Pillukat T, Hahn P, Müller LP, Spies CK (2015). Update TFCC: histology and pathology, classification, examination and diagnostics. Arch Orthop Trauma Surg.

[3] Möldner M, Unglaub F, Hahn P, Müller LP, Bruckner T, Spies CK (2015). Functionality after arthroscopic debridement of central triangular fibrocartilage tears with central perforations. J Hand Surg Am.

[4] Schädel-Höpfner M, Müller K, Gehrmann S, Lögters TT, Windolf J (2012). Therapy of triangular fibrocartilage complex lesions. Unfallchirurg.

[5] Frank U (2016). Untersuchung und MR-Morphologie des ulnokarpalen Handgelenkschmerzes. Handchir Scan.

[6] Nishizuka T, Tatebe M, Hirata H, Shinohara T, Yamamoto M, Iwatsuki K (2013). Simple debridement has little useful value on the clinical course of recalcitrant ulnar wrist pain. Bone Jt J.

[7] Palmer AK (1989). Triangular fibrocartilage complex lesions: a classification. J Hand Surg Am.

[8] Morrissy RT, Nalebuff EA (1979). Dislocation of the distal radioulnar joint: anatomy and clues to prompt diagnosis. Clin Orthop Relat Res.

[9] Seo JB, Kim JP, Yi HS, Park KH (2016). The outcomes of arthroscopic repair versus debridement for chronic unstable triangular fibrocartilage complex tears in patients undergoing ulnar-shortening osteotomy. J Hand Surg Am.

[10] Cooney WP, Linscheid RL, Dobyns JH (1994). Triangular fibrocartilage tears. J Hand Surg Am.

[11] Germann G, Harth A, Wind G, Demir E (2003). Standardisation and validation of the German version 2.0 of the disability of arm, shoulder, hand (DASH) questionnaire. Unfallchirurg.

[12] Hudak PL, Amadio PC, Bombardier C (1996). Development of an upper extremity outcome measure: the DASH (Disabilities of the Arm, Shoulder, and Hand). The Upper Extremity Collaborative Group (UECG). Am J Ind Med..

[13] Amirjani N, Ashworth NL, Olson JL, Morhart M, Chan KM (2011). Validity and reliability of the Purdue Pegboard Test in carpal tunnel syndrome. Muscle Nerve.

[14] Hermansdorfer JD, Kleinman WB (1991). Management of chronic peripheral tears of the triangular fibrocartilage complex. J Hand Surg Am.

[15] Löw S, Herold A, Eingartner C (2014). Standard wrist arthroscopy: technique and documentation. Oper Orthop Traumatol.

[16] Saito T, Malay S, Chung KC (2017). A systematic review of outcomes after arthroscopic débridement for triangular fibrocartilage complex tear. Plast Reconstr Surg.

[17] Arsalan-Werner A, Grüter L, Mehling IM, Moll W, Wölfle O, Sauerbier M (2018). Results after arthroscopic treatment of central traumatic lesions of the triangular fibrocartilage complex. Arch Orthop Trauma Surg.

[18] Cardenas-Montemayor E, Hartl JF, Wolf MB, Leclère FM, Dreyhaupt J, Hahn P, Unglaub F (2013). Subjective and objective results of arthroscopic debridement of ulnar-sided TFCC (Palmer type 1B) lesions with stable distal radio-ulnar joint. Arch Orthop Trauma Surg.

[19] Husby T, Haugstvedt JR (2001). Long-term results after arthroscopic resection of lesions of the triangular fibrocartilage complex. Scand J Plast Reconstr Surg Hand Surg.

[20] Osterman AL (1990). Arthroscopic debridement of triangular fibrocartilage complex tears. Arthroscopy.

[21] Wijffels M, Brink P, Schipper I (2012). Clinical and non-clinical aspects of distal radioulnar joint instability. Open Orthop J.

